# Polyphenol and Flavonoid Stability of Wild Blueberry (*Sideroxylon mascatense*) during Air- and Freeze-Drying and Storage Stability as a Function of Temperature

**DOI:** 10.3390/foods12040871

**Published:** 2023-02-17

**Authors:** Shaima Al Hasani, Zahir Al-Attabi, Mostafa Waly, Nasser Al-Habsi, Lyutha Al-Subhi, Mohammad Shafiur Rahman

**Affiliations:** Department of Food Science and Nutrition, College of Agricultural and Marine Sciences, Sultan Qaboos University, Muscat 123, Oman

**Keywords:** wild blueberry, physico-chemical, polyphenols, flavonoids, freeze drying, air drying

## Abstract

Būt (*Sideroxylon mascatense*) is an indigenous wild blueberry found in Oman. It has a very short season and is commonly preserved by drying. The aims of this study were to determine the physico-chemical characteristics and stability of phytochemicals (i.e., polyphenols and flavonoids) in the berries during drying (i.e., freeze-drying at −40 °C and air-drying at 60 and 90 °C) and the polyphenol stability of the dried berries as a function of storage temperature (i.e., 90, 70, 60, 40, 20, and −20 °C). The moisture content of fresh berry flesh was 64.5 g/100 g sample (wet basis). The crude protein and fat contents were higher in the seeds than in the flesh. Glucose and fructose were the main sugars and their concentrations were highest in the sample air-dried at 60 °C. The initial total polyphenol content (TPC) and total flavonoid content (TFC) of the flesh were 2.009 mg gallic acid equivalent (GAE)/g dry-solids and 0.199 mg catechin equivalent (CE)/g dry-solids, respectively. The samples air-dried at 90 °C and freeze-dried at −40 °C had higher TPC (i.e., 2.638 mg GAE/g dry-solids) and TFC (i.e., 0.395 mg CE/g dry-solids), respectively. There was a significant difference between the TPC and TFC of fresh and dried wild berries (*p* < 0.05). The freeze-dried wild berries retained a high TPC compared to the air-dried samples. The polyphenol storage stability of freeze-dried wild berries at different storage temperatures showed two phases: an initial release phase followed by a decay phase. The polyphenol storage stability was modeled using the Peleg model and the kinetic parameters were correlated with the storage temperature.

## 1. Introduction

*Sideroxylon mascatense* (*S. mascatense*) (synonym, *Monotheca buxifolia*) is a wild blueberry plant that belongs to the family Sapotaceae. The plant grows wild in the Hajar Mountains, north of Oman, and is locally called ‘Būt’, pronounced “boot”. The plant produces small, round, blue-colored fruit with seeds and they have a sweet-tangy taste when ripe [1,2]. The fruit is consumed fresh or dried. The fruit is expensive due to its seasonality and it is a challenge to harvest because it grows on the top of mountains [3]. The fruit and leaves contain antioxidant and free radical scavenging activities with health-promoting functional phenolic and flavonoid compounds [4,5,6].

Globally, there are many varieties of wild blueberries, which are distinct from one another in terms of color, size, flavor, chemical composition, and bioactive content [7]. Generally, berries are rich in phenolic compounds, which have good antioxidant properties [8]. They are seasonal fruit and need to be preserved to ensure their availability over the year. Drying and freezing are the most commonly used methods for their preservation.

Several traditional drying methods are used for berries, including hot air-drying, vacuum-drying, microwave-drying, and freeze-drying [9]. The effects of hot air-drying temperatures on berry quality and bioactive compounds have been studied over a wide range of temperatures, including 30, 40, 50, 60, 70, 80, and 90 °C [4,10,11]. Hot air-drying showed detrimental effects on the chemical constituents and physical characteristics of foods (i.e., structure, color, volume, density and porosity). This was due to the use of higher temperatures and longer drying times than vacuum-drying and microwave-drying [12]. In addition, crust formation, off flavors, and browning reactions (enzymatic and non-enzymatic) were also reported [10]. In contrast, several freeze-drying temperatures have been used, such as −20, −50, −40, and −60 °C [4,10]. The freeze-drying method showed high retention of functional bioactive compounds [13]; however, this process is costly and takes longer than other methods of drying. The authors of ref. [11] found that hot air-drying blueberries (*Vaccinium corymbosum* L.) at 60 °C for 23.4 h yielded lower levels of total phenolics and antioxidants compared to air-drying at 90 °C for 5.3 h. This was due to the longer drying time at 60 °C, which caused longer exposure to oxygen (i.e., oxidative damage). The prolonged exposure to the elevated temperature resulted in substantial degradation of the functional phenolic content. The authors of ref. [14] found air-drying of berries at 65 °C for 20 h gave a higher level of polyphenols than drying at 130 °C (i.e., 2 h) or 50 °C (i.e., 48 h) with better color retention. They pointed out that drying at 50 °C took longer, while drying at 130 °C took less time but the sample was exposed to very high temperature. Therefore, both temperature and exposure time affected the retention of polyphenols. However, drying time had more detrimental effects (e.g., degradation of antioxidants) at low temperature because of the formation of pores (i.e., higher porosity) during the longer drying time [10]. Higher porosity enhanced the diffusion of oxygen and caused oxidative damage. Therefore, long drying time (e.g., 48 h) at 50 °C caused more damage than at the intermediate temperature. However, higher temperatures (i.e., above 100 °C) caused the sample to collapse (i.e., low porosity), causing more negative effects on berry quality due to higher reaction rates at higher temperatures [14].

The authors of ref. [15] found that the total antioxidant activity of freeze-dried wild blueberry (*Vaccinium angustifolium*) powder decreased with increasing temperature from 25 to 80 °C. Ref. [16] studied the stability of phenolic compounds during storage (21, 4.4, and −20 °C for 8 weeks) of different products (i.e., ice pop, oatmeal bar, graham cracker cookie, juice, and gummy product) prepared with wild blueberry powder. The ice pop was the only product stored at −20 °C. The ice pop, oatmeal bar, and juice exhibited good phenolic compound stability. Generally, the phenolic compounds in berries existed in different forms of binding with the structure, thus their stability varied based on their free or bound form [14]. Therefore, more studies are needed to investigate the effects of different processing methods and storage on the functional components (e.g., total phenolic compounds (TPC) and total flavonoid content (TFC) stability and antioxidant activity) of berries [7].

On the other hand, fruit seeds are normally considered as waste. However, several fruit seeds can be utilized by the food and nutraceutical industries [17]. Unfortunately, negligible studies have reported on the bioactive components of berry seeds. They are expected to be a rich source of nutrients and to exhibit antioxidant capacity (i.e., health-beneficial properties). It was found that during the fermentation process of rabbiteye blueberries (*Vaccinium ashei*) for wine production, phenolics and flavonoids were released from both flesh and seeds, leading to significantly higher antioxidant activity [18]. Recently, interest in phenolic compounds in foods has reached new heights. Berries exhibit a wide range of therapeutic effects with potential benefits to human health due to the presence of natural antioxidants and their preventive effects against cancer, inflammation, allergies, diabetes and cardiovascular diseases [7,13,19]. The antioxidant and antitumor properties of wild blueberries (*Sideroxylon mascatense*) were presented by Al Hasani et al. (2021) [4].

There is a lack of published reports on the chemical composition, sensory characteristics, nutritional properties, and bioactive components of wild blueberries [1,5,7]. Therefore, this research aimed to assess the physico-chemical characteristics and phytochemical stability of *S. mascatense* under different drying methods (i.e., air- and freeze-drying) and polyphenol stability as a function of storage temperature (i.e., −20, 20, 40, 60, 70, and 90 °C).

## 2. Materials and Methods

### 2.1. Chemicals

Folin Ciocalteu reagent was purchased from Merck (Darmstadt, Germany). Sodium carbonate (7.5%), sodium hydroxide (1 M), sodium nitrite solution (5%), and aluminum chloride solution (10%) were of analytical grade (Sigma, St. Louis, MO, USA).

### 2.2. Būt Berry (Sideroxylon mascatense)

Būt berries (i.e., wild blueberries), grown in the mountains of Al-Jabal Al-Akhdar, Oman, were purchased at the time of harvesting (i.e., August). The samples were packed in commercial, zippered, plastic freezer bags (Brand; Falcon, 21 × 18 cm) and directly placed in a cold box with ice. The samples were transferred to the laboratory and stored at −60 °C until further analyses (i.e., 2 weeks).

### 2.3. Drying Methods

Two drying methods were applied in this study, i.e., air-drying and freeze-drying, which were used by Al Hasani et al. (2021) [4]. The frozen wild blueberries were thawed at room temperature and then pitted. For air-drying, the flesh and seeds were placed separately in a single layer on stainless steel trays and both were air dried at two different temperatures (i.e., 60 °C for 22 h and 90 °C for 8 h). These temperatures were selected since lower than 50 °C and above 100 °C previously showed more degradation of TPC [11,14]. These selected times were selected based on the desired final moisture of the dried wild blueberries. The target moisture content of the flesh was approximately 10.0 g/100 g sample, whereas the target for the seed was 3.0 g/100 g sample. The flesh was kept at higher moisture content due to the hygroscopic characteristics of the wild blueberry flesh. For freeze-drying, the flesh and seeds were frozen at −40 °C for 18 h and then freeze-dried for 5 days using a benchtop freeze-drying system (Catalogue Serial Number 7400030, Labconco, Kansas, MO, USA). The shelf temperature of the freeze-dryer was −40 °C and the chamber temperature was 20 °C (i.e., sample was frozen at −40 °C and then dried to 20 °C) with an operating pressure of 200 Pa. The condenser temperature was set at −80 °C. It was reported in the literature that commonly used freeze-drying temperatures are within −20 to −60 °C [4,10]. In this study, a middle temperature was considered for freeze-drying. The air-dried and freeze-dried samples were ground to fine powder using an electrical grinder (Philips HR2027, Hong Kong, China). The chemical composition, total polyphenols, and flavonoids were then analyzed in the flesh and seeds (i.e., before and after drying).

### 2.4. Chemical Composition

The chemical composition of the fresh and dried samples of both materials (i.e., flesh and seeds) were determined using the standard methods of the Association of Official Analytical Chemists [20]. The moisture content was determined by drying 2 g of sample in a controlled air oven at 105 °C for 18 h. The ash content was measured by burning of 2 g of sample in a muffle furnace at 600 °C for 5 h. The crude fat content was determined by extraction of 1 g of sample with petroleum ether at 60 °C for 8 h using a soxhlet apparatus. The crude protein content was measured by the Kjeldahl method using a Foss-2300 Kjeltec analyzer unit (Foss, Hoganas, Sweden). In this method, 0.5 g of dried sample was digested in sulfuric acid at 420 °C for 3 h using selenium as a catalyst and then distilled using sodium hydroxide. Finally, the carbohydrate content was determined by calculating the difference between 100 and the sum of the moisture, protein, lipid, and ash contents in g/100 g sample. A sample calculation is presented in the Results and Discussion section.

### 2.5. Sugar Analysis

The sugar composition (i.e., glucose and fructose) was determined by high performance liquid chromatography (HPLC) coupled with a refractive index detector (RID), according to Fu et al. (2015) [21]. A sample (0.5 g) of fruit homogenate was dissolved in 100 mL of distilled water. The solution was centrifuged at 6000 rpm for 20 min at 4 °C and the supernatant was filtered through a 0.45 μm membrane filter (Millipore Millex-HV Hydrophilic PVDF, Millipore, Burlington, MA, USA) before HPLC analysis. The mobile phase was acetonitrile/water (75:25, *v*/*v*). The flow rate was 1 mL/min, while the injection volume was 10 µL. The column was SUPEL COSIL LC-NH2 HPLC (Sigma-Aldrich, St. Louis, MO, USA). Calibration curves were prepared using different concentration of glucose and fructose standards (0.01, 0.05, 0.1, 0.2, 0.5, and 1 g in 100 mL).

### 2.6. Titratable Acidity and pH

The titratable acidity (TA) was determined as described by Fu et al. (2015) [21]. The fruit filtrate of 2 g was titrated against standardized 0.1 mol/L NaOH solution after adding 2–3 drops of phenolphthalein as the indicator. The results were expressed as g/100 g of sample. The pH of the wild blueberry filtrate was measured using a calibrated pH meter (Delta 320-S, Mettler Toledo, Shanghai, China).

### 2.7. Preparation of Fruit Extract for Phytochemical Analysis

Each powder (1 g) (i.e., fresh and dried samples of both flesh and seeds) was extracted in 100 mL of distilled water at 20 °C for 2 h with agitation using a magnetic stirrer (J. Bibby Science products limited, Stone, Staffordshire, UK). Water was used to extract the phenolics and flavonoids instead of other solvents (e.g., ethanol and methanol) due to its low cost without the need for any chemical solvents. The extracts were then filtered and centrifuged at 6000 rpm for 20 min at 4 °C. The supernatants were collected in dark plastic bottles and the crude extracts were analyzed.

### 2.8. Total Phenolic Content (TPC)

TPC was assessed using the Folin-Ciocalteu assay of Singleton and Rossi (1965) [22] with slight modifications. Folin-Ciocalteu reagent (250 µL) was mixed with sample extract and incubated for 5 min. Then, 750 µL of sodium carbonate solution (1.9 M) was added and the resulting solution was incubated in the dark for 2 h at room temperature. The absorbance of the mixture was measured at 765 nm using an UV-Vis Perkin Elmer Lambda 25 double beam spectrophotometer and compared with that of gallic acid standards. The concentration of phenolic compounds in the fruit extract was expressed as mg gallic acid equivalent (GAE) per g dry matter. Each measurement was performed thrice.

### 2.9. Total Flavonoid Content (TFC)

TFC of the extracts was determined according to the aluminum chloride colorimetric assay [23]. Distilled water (4 mL) was added to the fruit extract and then 5% sodium nitrite solution was added, followed by 10% aluminum chloride solution (0.3 mL). The test tube was incubated at room temperature for 5 min and then 2 mL of 1 M sodium hydroxide was added to the mixture. The mixture was then vortexed and the absorbance was measured at 510 nm. TFC in the fruit was expressed as mg catechin equivalent (CE) per g dry matter.

### 2.10. Storage Stability of Polyphenols

The degradation of the phenolic compounds in freeze-dried flesh was investigated at different storage temperatures (i.e., −20, 20, 40, 60, 70, and 90 °C). These temperatures were selected considering freezer temperature (i.e., −20 °C), room temperature (20 °C), and elevated temperatures (40, 60, 70, and 90 °C). Freeze-dried sample was divided into 6 g portions and kept in closed bottles. Samples were stored at −20 °C and 20 °C for 5 months. Other samples were stored at 40, 60, 70, and 90 °C for 63, 14, 14, and 10 days, respectively. Samples stored at −20, 20, and 40 °C were analyzed for TPC at 0 and 3 days and then on weekly basis. Samples stored at 60 and 70 °C were analyzed after half a day and then daily for up to 14 days. Samples stored at 90 °C were analyzed every 2 h until 15 h and then daily until 10 days.

### 2.11. Storage Stability Prediction Model

Based on a peak in the reaction kinetics (i.e., concentration-time curve), Peleg (1996) [24] proposed a model considering the formation and decay of active bioactive components as:

(1) $$C=Y_{1}\times Y_{2}$$

where *Y*_1_ and *Y*_2_ are the concentration kinetics in the formation (i.e., before inflection point) and decay (i.e., after inflection) periods, respectively [25]. The formation kinetics, *Y*_1_, were modeled by the shifted logistic function as:

(2) $$Y_{1}=\frac{a}{1+exp\left[k_{g}\left(t_{g}-t\right)\right]}$$

where *a* is the peak concentration (mg/g dry solid (ds)), *t_g_* is the model parameter (h), and *k_g_* is the rate constant of release of the bioactive component (h^−1^). The decay kinetics, *Y*_2_, were modeled by the Fermi distribution function as:

(3) $$Y_{2}=\frac{1}{1+exp\left[k_{d}\left(t-t_{d}\right)\right]}$$

where *k_d_* (h^−1^) and *t_d_* (h) are the decay rate constant and model parameter, respectively. The activation energies of the release and decay periods were estimated using the Arrhenius equation as:

(4) $$k=A\left[exp\left(\frac{\Delta E}{R\;T}\right)\right]$$

where *k* is the rate constant of the release or decay phases (i.e., *k_g_* or *k_d_*), ∆*E* is the activation energy (J/mole), *R* is the universal gas constant (8.314 J/mole K), and *T* is the storage temperature (K).

### 2.12. Statistical Analysis

Results of all replicated chemical analyses are expressed as the mean ± standard deviation. Differences among chemical compositions due to different drying methods were analyzed using the Least Significant Difference (LSD) test at a significance level of *p* < 0.05 using Graph Pad Prism version 5.03 software (GraphPad Software Inc. San Diego, CA, USA). The model parameters were estimated considering non-linear regression using the NLR procedure in NLREG software (Phillip Sherrod, Brentwood, TN, USA) [26].

## 3. Results and Discussion

### 3.1. Chemical Composition

The chemical composition of the wild blueberries (*S. mascatense*) is presented in Table 1 and Table 2 for the flesh and seeds, respectively, at different drying conditions. The moisture contents of the fresh flesh and seeds were 64.5 and 32.9 g/100 g sample (wet basis), respectively (Table 1 and Table 2). Similarly, Brauch et al. (2016) [27] reported that fresh maqui (*Aristotelia chilensis* (Mol.) *Stuntz*) berries contained a moisture content of 61.8 g/100 g sample. However, a higher moisture content (85.78 g/100 g sample) was reported for fresh rabbiteye blueberries by Reque et al. (2014) [8]. Differences in the moisture content of berries could be due to their growing conditions (i.e., climate and rainfall), cultivar, maturity stage, and storage condition [28]. In addition, the hot air-dried samples (8.6–10.9 g/100 g sample) of the wild blueberries in this study had very low moisture contents compared to rabbiteye blueberries (19.6 g/100 g sample).

The ash content of the flesh of the fresh wild blueberries was 2.8 g/100 g ds. A significant difference in the ash content was observed as a result of the different drying processes. The ash content increased from 2.8 g/100 g ds in the freeze-dried sample to 3.2 and 3.3 g/100 g ds in the air-dried samples at 60 and 90 °C, respectively. These values were higher than that reported by Reque et al. (2014) [8] for rabbiteye blueberries (i.e., 1.2 g/100 g ds). The variation in results could be due to differences in harvesting time for blueberries and the mineral concentration in the soil [8]. On the other hand, the seeds had a lower ash content than the flesh, with no significant difference between fresh and dried seeds.

The protein content of the fresh flesh of the wild blueberries was 1.90 g/100 g ds. In contrast, Reque et al. (2014) [8] detected a higher protein content for rabbiteye blueberries (4.10 g/100 g ds). The highest protein content in the current samples of wild blueberries was found in the sample dried at the highest temperature (90 °C). However, no significant difference in protein content was found between different drying temperatures (*p* < 0.05), indicating the thermal stability of the proteins in blueberries [8]. The wild blueberry seeds contained more protein (6.15 g/100 g ds) than the flesh. This clearly indicated that the seeds might be a good source of protein.

The fat content of the fresh flesh of the wild blueberries was 5.04 g/100 g ds, which was higher than that reported for rabbiteye blueberries for both fresh (0.73 g/100 g ds) and a sample air-dried at 80 °C for 6 h (0.42 g/100 g ds) [8]. However, in the current study, there was a significant decrease in fat content from fresh to dried samples, with the highest loss of lipids at 90 °C. The high drying temperature can cause the melting of lipids, which could drip out of the blueberries onto the metal tray [29]. It was observed that a sticky portion remained on the tray, which may have contained a portion of the fat. Oxidation of fat could also cause a reduction in the amount of fat recovered. Overall, the seeds of the wild blueberries were a richer source of fat (9.64 g/100 g ds) than the flesh.

The fresh flesh of the wild blueberries had a high carbohydrate content (90.30 g/100 g ds). This was calculated from the difference between 100 and the sum of the moisture, protein, lipid, and ash contents in g/100 g sample (i.e., wet basis). For example, the moisture, ash, protein, and fat contents were 64.5 g, 0.994 g (i.e., (2.8 × 35.5/100) g), 0.675 g (i.e., (1.9 × 35.5/100) g), and 1.775 g (i.e., (5.0 × 35.5/100) g), respectively (Table 1, column 1). The carbohydrate content was calculated as 32.06 g (i.e., 100 − (64.5 + 0.994 + 0.675 + 1.77)) (Table 1, Column 1), converting it into dry solids as 90.30 g/100 ds (i.e., (32.06/(100 − 64.5)) × 100). Although less than in the current study, fresh maqui berries also had a significant amount of carbohydrates (86.2 g/100 g ds) [29]. Both studies reported a consistently lower amount of carbohydrates in dried than in fresh samples. In addition, the seeds had a good level of carbohydrates. The fresh seeds contained 82.80 g/100 g ds carbohydrates and the value was reduced as the drying temperature increased, reaching 78.52 g/100 g for the sample air-dried at 90 °C.

### 3.2. Sugars

Glucose and fructose are the key sugars in berries besides sucrose [30]. In the present study, glucose and fructose were detected in the wild blueberry samples. There was a significant difference in the glucose content among all samples, but not in the fructose content. The amount of glucose (37.39 g/100 g ds) was higher than the amount of fructose (31.52 g/100 g ds) (Table 1). Dried rabbiteye blueberries were found to contain 54.34 g/100 g ds of reducing sugars (i.e., glucose and fructose) [8], which was less than the amount reported in the current study. Differences in sugar levels are attributed to maturity stage and genetics [30]. In addition, the pre-harvest environment, including temperature, solar radiation, irrigation, water availability, soil mineral content, fertilization regime, and pruning techniques, could also influence fruit sugar levels [21]. The highest concentrations for both sugars reached 45.99 g/100 g ds of glucose and 32.84 g/100 g ds of fructose in the sample air-dried at 60 °C. Thereafter, sugar levels decreased with increasing air drying temperature up to 90 °C. The reduction in the sugar content in plant materials during drying could be attributed to the non-enzymatic browning reaction, especially at high drying temperatures [31].

### 3.3. Titratable Acidity and pH

Table 1 and Table 2 show that changes in TA were not significant in both the flesh and seeds (*p* > 0.05). Generally, the TA in seeds (0.20 g/100 g) was higher than that in the fresh flesh (0.13 g/100 g) subjected to different drying methods. The authors of ref. [8] found that the pH of fresh rabbiteye blueberries was 2.92, while dried samples had a pH of 2.82. Indeed, the pH values in the present study were higher than those previously reported, but they still indicated that the wild blueberries could be considered acidic. The seeds of the freeze-dried sample at −20 °C exhibited a high pH (7.32), which may influence its sensory attributes. Ref. [32] reported that bioactive compounds were stable at pH values between 1 and 4, while anthocyanins were generally degraded at pH 7.

### 3.4. Total Polyphenol and Flavonoid Content for Fresh and Dried Blueberries

The TPC and TFC of the wild blueberries used in this study for both flesh and seed are presented in Table 3. The flesh of the fresh sample had 2.009 mg GAE/g ds (i.e., 1.221 mg GAE/g fresh sample) compared to 0.069 mg GAE/g ds in the fresh seeds. Ref. [33] found a higher TPC (3.66–4.57 mg GAE/g fresh weight) in wild blueberries (*Vaccinium myrtillus* L.). Additionally, ref. [34] reported an average of 2.86 mg GAE/g TPC in wild blueberries. A higher TPC (9.44 mg GAE/g ds) was reported by ref. [35] in blueberries. However, the current findings indicated that wild blueberries (*S. mascatense*) can be a good source of phenolic compounds according to the classification reported by Vasco et al. (2008) [36]: low <1 mg GAE/g, medium 1–5 mg GAE/g, and and high >5 mg GAE/g.

The TFC of the fresh flesh sample was 0.199 mg CE/g ds (i.e., 0.121 mg CE/g fresh sample) (Table 3). Variations in reported TPC and TFC values in blueberries could be due to differences in cultivar, ripening stage, weather and soil conditions, as well as the solvent extraction method [5,33].

The TPC of the freeze-dried sample was 2.317 mg GAE/g ds, which increased with increasing drying temperature, reaching a maximum at 90 °C (2.638 mg GAE/g ds) (Table 3). Ref. [14] reported a higher TPC in berries dried at 65 °C compared to freeze-dried berries and those air-dried at 50 °C. Similarly, ref. [12] observed a decrease in TPC after air drying at 40 °C, while it increased when air drying at 60 and 80 °C. The authors mentioned that this could be linked to the temperature-dependent release of bound polyphenols. In the case of chamomile flower, ref. [37] observed high TPC after freeze-drying, while no significant difference in TPC was observed between air-drying at 20 or 40 °C and drying at 80 °C resulted in a significantly decreased the TPC. Ref. [38] found that willow leaves dried below 50 °C had a significantly higher TPC than those dried at 90 °C. The polyphenolic compounds exist in free, soluble ester, and insoluble bound forms, which are susceptible to heat [39]. Insoluble bound phenolics are released upon damage of cellular constituents. At the same time, the disruption of cell walls releases oxidative enzymes that can accelerate the release of bound polyphenols [12]. The release of phenolics also depends on the evaporation of polyphenols, which could be related to their binding with other components [38].

The TFC of the freeze-dried flesh of the wild blueberries (i.e., *S. mascatense*) at −40 °C was 0.395 mg CE/g ds. The TFC was significantly decreased in samples air-dried at 60 and 90 °C, reaching values of 0.195 and 0.186 mg CE/g ds, respectively (*p* < 0.05). Ref. [40] reported that thermally induced cellular damage released bound bioactive compounds and these were further damaged by oxygen, enzymes, and light. The fresh seeds contained 0.056 mg CE/g ds. The TFC values of the seeds at −40, 60, and 90 °C were 0.089, 0.044, and 0.039 mg CE/g ds, respectively (Table 3). The TFC was significantly increased in the case of the freeze-dried sample and air drying resulted in a decrease in the TFC (*p* < 0.05). The reverse trends of TPC and TFC during drying could be due to different binding natures and thermo-labile variability [41], interference of other phenolic compounds [42], and enzyme activities [43]. Therefore, the drying method needs to be selected based on the target bioactive components, as also pointed out by the authors of ref. [42].

### 3.5. Stability of Phenolic Compounds

**Figure 1:**
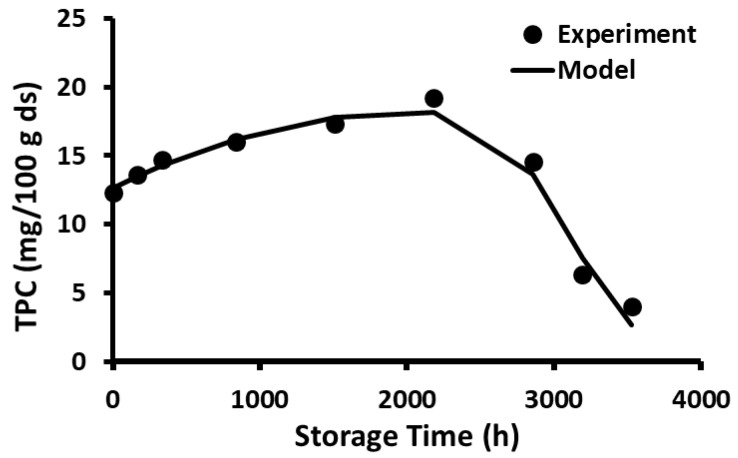
Polyphenol content during storage at −20 °C for 5 months.

**Figure 2:**
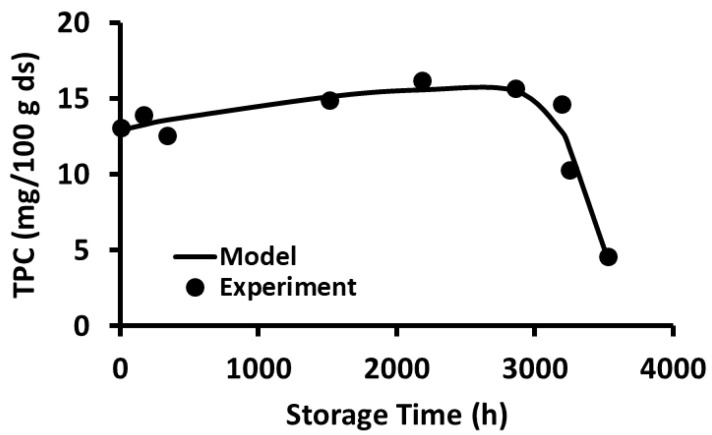
Polyphenol content during storage at 20 °C for 5 months.

**Figure 3:**
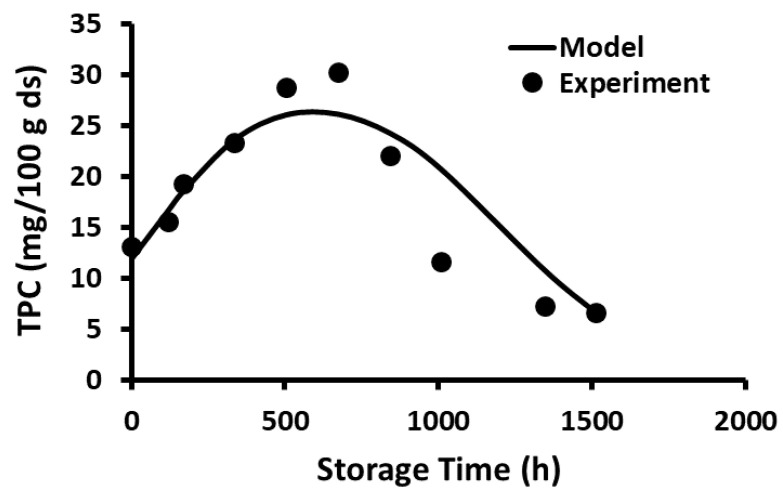
Polyphenol content during storage at 40 °C for 63 days.

**Figure 4:**
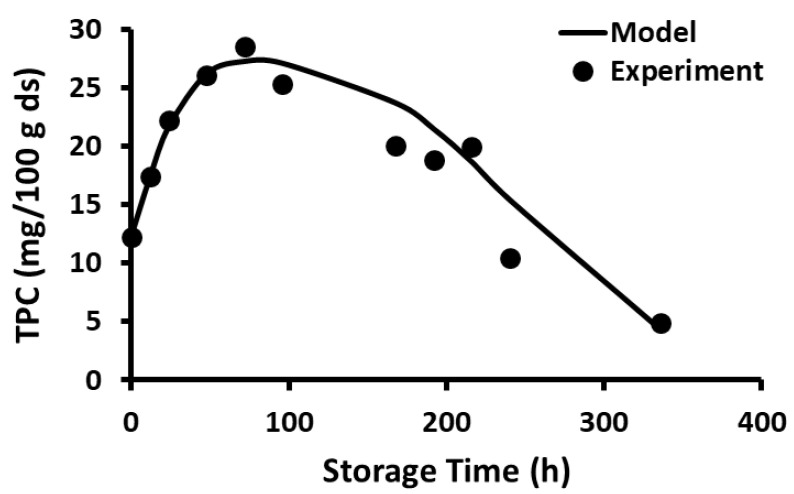
Polyphenol content during storage at 60 °C for 14 days.

**Figure 5:**
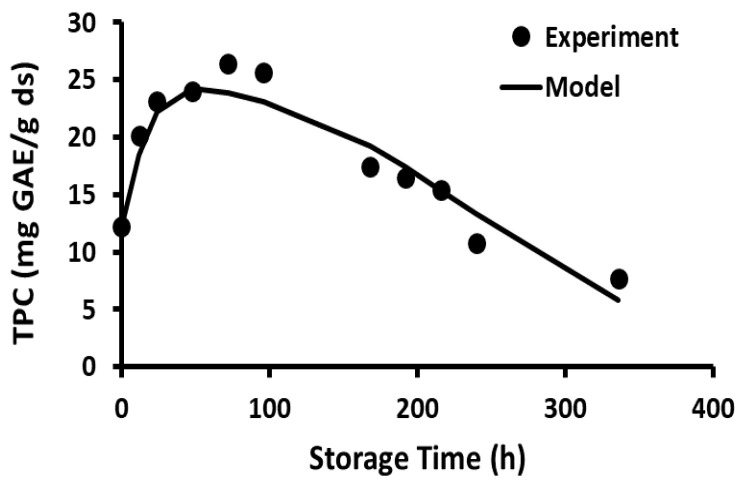
Polyphenol content during storage at 70 °C for 14 days.

**Figure 6:**
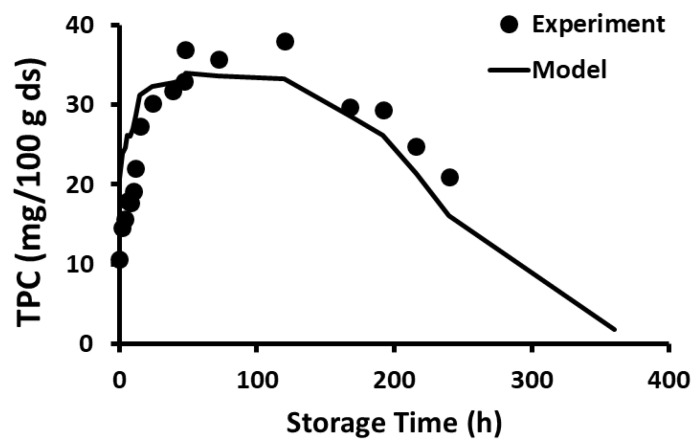
Polyphenol content during storage at 90 °C for 10 days.

The stability of polyphenols in the freeze-dried wild blueberries as a function of storage time and temperature is shown in Figure 1, Figure 2, Figure 3, Figure 4, Figure 5 and Figure 6. Initially, TPC increased (i.e., release phase) with storage time and reached a peak, followed by a sharp decay (decay phase) (Figure 1). The two observed phases were due to the presence of different forms of bound and free polyphenols in the blueberries, and release was dependent on the treatment [44,45]. In the case of vitamin C stability in fresh broccoli stored at 4 °C, the initial peak was observed at 2 days followed by decay [46]. Ref. [47] studied the polyphenol stability of brined olives treated with different salts and ozone, observing two phases in stability (i.e., release and decay phases). However, these authors only studied stability at room temperature. Most phenolic compounds in berries exist in a variety of conjugated forms, either with sugars via O-glycosidic bonds or with other polyols as esters [45]. Ref. [48] studied the TPC stability of cherry fruit extracts at 2, 22, 55, and 75 °C and showed first order decay kinetics. These authors observed only decay since the extracts mainly contained free polyphenols. A similar observation was reported for the polyphenol anthocyanin in carrot juice stored at 94–37 °C [49].

The current study showed that the release of polyphenols dominated in the first phase compared to the rate of decay, while the decay of polyphenols dominated in the second phase compared to the rate of release. In the case of storage at −20 and 20 °C, the release phase was much slower than the decay phase (Figure 1 and Figure 2), while the release phase was faster than the decay phase at temperatures at and above 40 °C (Figure 3, Figure 4, Figure 5 and Figure 6). These behaviors could be due to the temperature-dependent release of bound polyphenols and decay of free polyphenols.

**Figure 7:**
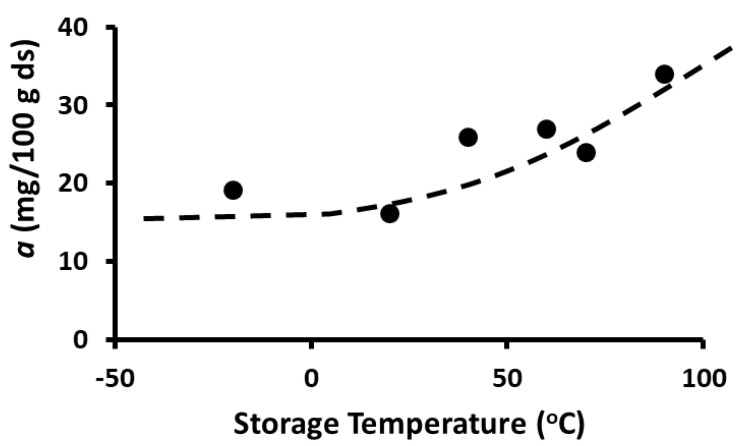
Peak polyphenol concentration as a function of storage temperature (line is a trend guide).

**Figure 8:**
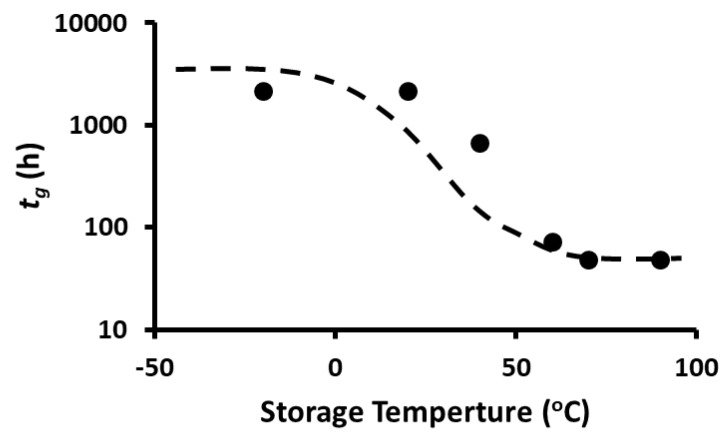
Peak time as a function of storage temperature (line is a trend guide).

**Figure 9:**
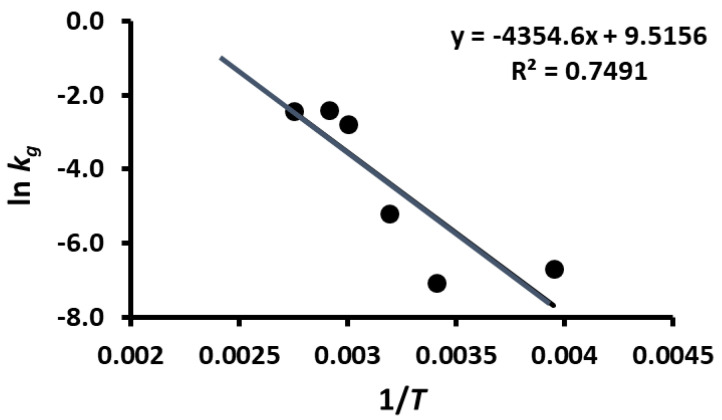
Plot of ln *k_g_* as a function of 1/*T*.

**Figure 10:**
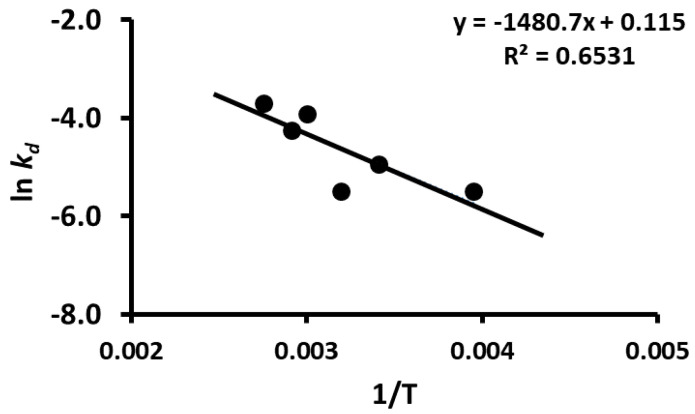
Plot of log *k_d_* as a function of 1/*T*.

TPC stability was modeled by Equation (1) and the parameters were determined by non-linear regression. The peak concentration (i.e., model parameter *a*) as a function of temperature is shown in Figure 7 and it remained nearly the same during storage at 20 and −20 °C, while it increased at higher temperatures (i.e., 40–90 °C). However, peak time (i.e., model parameter *t_g_*) also remained nearly the same during storage at 20 and −20 °C but then decreased at temperatures up to 60 °C, followed by an equilibrium period (Figure 8). The rate constants of the release and decay phases increased with the increase in storage temperature. These were plotted as ln *k_g_* or ln *k_d_* as a function of 1/*T*, as shown in Figure 9 and Figure 10 (i.e., Arrhenius plots). The activation energies were estimated as 36.2 kJ/mole and 12.3 kJ/mole for the release and decay phases, respectively. It was observed that the activation energy was nearly 3 times higher for the release phase than for the decay phase. This indicated that more energy was required to release the bound polyphenols. Overall, 20 and −20 °C were the best storage temperatures for retaining a high TPC. However, comparing the refrigeration costs of low temperature storage and simplicity, storing dried wild blueberries at room temperature (i.e., 20 °C) could be recommended.

## 4. Conclusions

The current work highlighted the compositional characteristics of wild blueberry (*Sideroxylon mascatense*) flesh and seeds. The seeds were a rich source of protein and fat compared to flesh. The fat content was significantly higher in freeze-dried samples for both flesh and seeds. TPC and TFC were higher in dried than in fresh wild blueberries. The TPC was higher in the sample air-dried at 90 °C, which was higher than that of the freeze-dried sample. However, the freeze-dried sample retained a higher TFC than the air-dried sample. Therefore, the drying method should be selected based on the target bioactive components. The storage stability of polyphenols showed two-phase kinetics, including release and decay phases with a peak. The peak concentration and time to reach the peak concentration remained constant at and below 20 °C, whereas the peak concentration increased and the peak time decreased above 20 °C. The activation energy was nearly 3 times higher for the release phase than for the decay phase (i.e., more energy was involved in the release of bound polyphenols). Dried wild blueberries need to be stored at 20 or −20 °C; however, storage at 20 °C is recommended based on refrigeration costs and simplicity.

## Figures and Tables

**Table 1:** Chemical composition of wild blueberry (*Sideroxylon mascatense*) flesh.

Component	Fresh	Freeze-Dried −40 °C	Air-Dried 60 °C	Air-Dried 90 °C
Moisture ^1^	64.5	11.9	10.9	8.6
Moisture ^2^	181.7 ± 5.4	13.54 ± 0.09	12.17 ± 0.31	9.43 ± 0.09
Ash ^2^	2.8 ± 0.1 ^a^	2.78 ± 0.01 ^a^	3.19 ± 0.14 ^bc^	3.34 ± 0.14 ^cd^
Protein ^2^	1.9 ± 0.5 ^a^	4.71 ± 0.09 ^c^	4.82 ± 0.11 ^c^	4.92 ± 0.92 ^c^
Fat ^2^	5.0 ± 1.3 ^a^	3.56 ± 0.35 ^ac^	3.07 ± 0.64 ^cd^	2.41 ± 0.19 ^d^
Carbohydrates ^2^	90.30	88.95	90.01	89.33
Glucose ^2^	37.4 ± 0.6 ^a^	40.82 ± 0.68 ^bc^	45.99 ± 0.82 ^cd^	41.01 ± 1.21 ^ad^
Fructose ^2^	31.5 ± 0.2 ^a^	30.84 ± 0.61 ^a^	32.84 ± 0.41 ^a^	30.32 ± 0.82 ^a^
Titratable Acidity ^3^	0.13 ± 0.06 ^a^	0.10 ± 0.00 ^a^	0.17 ± 0.06 ^a^	0.10 ± 0.00 ^a^
pH	4.91 ± 0.01 ^a^	6.35 ± 0.02 ^b^	5.42 ± 0.02 ^c^	5.43 ± 0.02 ^c^

Note: Data are expressed as mean ± standard deviation of triplicate samples. Different letters within a row indicate that values are significantly different (*p* < 0.05). ^1^ Wet basis (g/100 g sample). ^2^ g/100 g dry solids. ^3^ Percentage.

**Table 2:** Chemical composition of wild blueberry (*Sideroxylon mascatense*) seeds.

Component	Fresh	Freeze-Dried −40 °C	Air-Dried 60 °C	Air-Dried 90 °C
Moisture ^1^	32.9	3.18	3.19	3.07
Moisture ^2^	49.0 ± 1.7	3.29 ± 0.06	3.31 ± 0.08	3.16 ± 0.60
Ash ^2^	1.4 ± 0.2 ^a^	1.33 ± 0.11 ^a^	1.27 ± 0.1 ^a^	1.38 ± 0.03 ^a^
Protein ^2^	6.2 ± 0.7 ^a^	7.70 ± 0.30 ^b^	6.99 ± 0.27 ^ac^	5.83 ± 0.49 ^ad^
Fat ^2^	9.6 ±1.5 ^a^	17.15 ± 0.21 ^b^	15.56 ± 0.16 ^c^	14.27 ± 0.16 ^d^
Carbohydrates ^2^	82.80	73.82	76.18	78.52
Titratable Acidity ^3^	0.2 ± 0.01 ^a^	0.13 ± 0.06 ^a^	0.17 ± 0.06 ^a^	0.15 ± 0.05 ^a^
pH	4.4 ± 0.2 ^a^	7.32 ± 0.03 ^b^	5.04 ± 0.03 ^c^	5.91 ± 0.05 ^d^

Note: Data are expressed as mean ± standard deviation of triplicate samples. Different letters within a row indicate that values are significantly different (*p* < 0.05). ^1^ Wet basis (g/100 g sample). ^2^ g/100 g dry solids. ^3^ Percentage.

**Table 3:** Total polyphenol and total flavonoid content of wild blueberries (*Sideroxylon mascatense*).

	Drying Methods				
Bioactive Compounds	Material	Fresh	Freeze-Dried −40 °C	Air-Dried 60 °C	Air-Dried 90 °C
TPC (mg GAE/g ds)	Flesh	2.009 ± 0.012 ^a^	2.317 ± 0.005 ^b^	2.486 ± 0.004 ^c^	2.638 ± 0.033 ^d^
	Seed	0.069 ± 0.001 ^a^	0.117 ± 0.002 ^b^	0.623 ± 0.007 ^c^	0.632 ± 0.002 ^c^
TFC (mg CE/g ds)	Flesh	0.199 ± 0.001 ^a^	0.395 ± 0.004 ^b^	0.195 ± 0.000 ^c^	0.186 ± 0.003 ^d^
	Seed	0.056 ± 0.001 ^a^	0.089 ± 0.000 ^b^	0.044 ± 0.000 ^c^	0.039 ± 0.000 ^d^

Note: Data are expressed as mean ± standard deviation of triplicate samples. Different letters within a row indicate that values are significantly different (*p* < 0.05).

## Data Availability

The data are available from the corresponding author.
